# Better self-care through co-care? A latent profile analysis of primary care patients' experiences of e-health–supported chronic care management

**DOI:** 10.3389/fpubh.2022.960383

**Published:** 2022-09-23

**Authors:** Carolina Wannheden, Marta Roczniewska, Henna Hasson, Klas Karlgren, Ulrica von Thiele Schwarz

**Affiliations:** ^1^PROCOME, Medical Management Center, Department of Learning, Informatics, Management and Ethics, Karolinska Institutet, Stockholm, Sweden; ^2^Psychology Department, SWPS University of Social Sciences and Humanities, Sopot, Poland; ^3^Unit for Implementation and Evaluation, Center for Epidemiology and Community Medicine, Stockholm Region, Stockholm, Sweden; ^4^MINT, Department of Learning, Informatics, Management and Ethics, Karolinska Institutet, Stockholm, Sweden; ^5^SimArena, Department of Health and Functioning, Faculty of Health and Social Sciences, The Western Norway University of Applied Sciences, Bergen, Norway; ^6^Department of Research, Education, Development and Innovation, Education Center, Södersjukhuset, Stockholm, Sweden; ^7^School of Health, Care and Social Welfare, Mälardalen University, Västerås, Sweden

**Keywords:** person-centered, patient experience, role clarity, self-efficacy, e-health, co-production, latent profile analysis, self-care

## Abstract

**Background:**

Efficient self-care of chronic conditions requires that an individual's resources be optimally combined with healthcare's resources, sometimes supported by e-health services (i.e., co-care). This calls for a system perspective of self-care to determine to what extent it involves demanding or unnecessary tasks and whether role clarity, needs support, and goal orientation are sufficient. This study aims to explore typical configurations of how the co-care system is experienced by individuals with chronic conditions who used an e-health service supporting self-monitoring and digital communication with primary care.

**Method:**

We performed a latent profile analysis using questionnaire data from two waves (7 months apart) involving 180 of 308 eligible patients who pilot-tested an e-health service for co-care at a Swedish primary care center. The five subscales of the Distribution of Co-Care Activities (DoCCA) scale were used to create profiles at Time 1 (T1) and Time 2 (T2). Profiles were described based on sociodemographic variables (age, gender, education level, and health condition) and compared based on exogenous variables (self-rated health, satisfaction with healthcare, self-efficacy in self-care, and perceptions of the e-health service).

**Results:**

We identified four typical configurations of co-care experiences at T1: strained, neutral, supportive, and optimal. Patients with optimal and supportive profiles had higher self-rated health, self-efficacy in self-care, and satisfaction with healthcare than patients with strained and neutral profiles. Slightly more than half transitioned to a similar or more positive profile at T2, for which we identified five profiles: unsupportive, strained, neutral, supportive, and optimal. Patients with optimal and supportive profiles at T2 had higher self-efficacy in self-care and satisfaction with healthcare than the other profiles. The optimal profiles also had higher self-rated health than all other profiles. Members of the optimal and supportive profiles perceived the effectiveness of the e-health service as more positive than the unsupportive and strained profile members.

**Discussion:**

Primary care patients' co-care profiles were primarily distinguished by their experiences of needs support, goal orientation, and role clarity. Patients with more positive co-care experiences also reported higher self-rated health, self-efficacy in self-care, and satisfaction with healthcare, as well as more positive experiences of the e-health service.

## Introduction

Chronic conditions such as cancer, cardiovascular diseases, chronic respiratory problems, and diabetes are major causes of disability and the leading causes of death across OECD countries, where more than one-third of people aged 16 and older experience at least one long-term condition or health problem (1). For the individual, managing a chronic condition involves multiple behaviors aiming to achieve, maintain, or promote health. Such behaviors may include identifying and monitoring symptoms and key health parameters; planning, following, and adjusting treatment regimens; and coordinating resources to support self-care (2). Healthcare services may be one of several resources that matter to individuals. Accordingly, support for self-care is a critical component in healthcare models such as the Chronic Care Model (3), reflecting that self-care is being recognized as the new principal source of care (4). Support for self-care is offered through interaction between healthcare professionals and the individual. It can also entail the provision of e-health services (i.e., health services and information that are delivered or enhanced through the internet and related technologies) (5).

The boundaries between healthcare and self-care are not sharply defined when it comes to chronic conditions. Some researchers define self-care as activities that are planned together with healthcare, which per definition implies an overlap between an individual and a healthcare provider's responsibilities (2). Self-care of chronic conditions involves behaviors to maintain or improve physical and emotional stability (i.e., self-care maintenance), monitoring changes in signs and symptoms (i.e., self-care monitoring), and responding to signs and symptoms when they occur (i.e., self-care management) (6). The continuously rising availability and adoption of digital health technologies to support individuals in their self-care activities and communication within healthcare further blurs the boundaries between self-care and healthcare (7). For example, technology can enable individuals to take on activities traditionally performed by healthcare professionals, such as performing and monitoring blood pressure measurements (8), thereby enabling timely interventions. These developments call for a system perspective of chronic care management that acknowledges that more than one actor can perform the necessary activities.

Whereas it is frequently recognized that self-care of chronic conditions often involves interaction with healthcare, with or without the support of technology, less attention has been paid to how this system of actors (the individual, healthcare professionals, and e-health services) can be conceptualized and optimized. Co-care has been introduced as a construct that views chronic care management as a system, emphasizing that the role of healthcare professionals and e-health services is to complement people's personal resources in managing their health to achieve desired health outcomes (9). Thus, the co-care concept highlights a system perspective of self-care of chronic conditions. An optimal co-care system is oriented toward the goals of an individual living with the chronic condition. It supports the individual's needs and distributes the chronic care management activities that need to be performed so that the individual perceives the demands and tasks as reasonable and legitimate and that the roles of those involved are clear (10). This definition acknowledges that individuals may have different preferences for distributing chronic care management activities between themselves, others (including healthcare professionals), and e-health services. However, the ways individuals with chronic conditions perceive their co-care system has yet to be investigated.

A rich body of literature has aimed to assess patients' experiences of self-care and healthcare. For example, studies have assessed patient experiences in relation to a certain provider or healthcare professional (11, 12). Studies have also focused on the experience of specific e-health services (13). Others offer insight into the ways certain care episodes or activities are perceived, such as the decision-making process (14), or the way people experience choosing between different options (15). For example, a systematic review of barriers and facilitators of shared decision-making with older people showed that making shared decisions is facilitated when patients are encouraged to share their valued goals (16). Studies have also examined the ways patients perceive chronic care management (17), but the focus has been on how patients experience the activities that healthcare performs, not how well healthcare functions as a resource for the individual. This reflects an “inside-out” perspective on patient experiences (18), where the individual is defined through their relation to healthcare (i.e., as a patient). A need exists for a perspective that acknowledges that the management of a chronic condition includes activities that could be done by either the individual (i.e., self-care) or others (e.g., healthcare professionals). A recent review found that patients' experiences of collaborative care practices were closely linked to their role and suggested that further investigation into the patient role and its influence on experiences is necessary to identify improvement strategies for collaborative care frameworks (19). Thus, although the ways individuals perceive specific parts of chronic care management in self-care and healthcare have been investigated, the system perspective whereby chronic care management is approached as a system of activities that takes place around the clock and that can be performed by various actors, including e-health services (9), remains largely unexplored. Such knowledge is needed for the optimization of the chronic care management system (i.e., the distribution of tasks so that the individuals for whom the system exists benefit).

Furthermore, individuals with chronic conditions are a heterogeneous group, with different experiences, needs, and expectations related to both self-care and healthcare. For example, health status or cognitive functioning affects the ability of older adults with multiple chronic conditions to participate in shared decision-making (16). In addition, large variation has been found in the degree to which patients with chronic heart diseases understand and perceive their activity data from a wearable tracker, spanning from it being a cognitive and emotional resource to causing confusion, uncertainty, and fear (20). Thus, the same technology can lead to very different experiences, and in the end, have different effects on people's experienced ability and confidence to perform self-care. It remains to be investigated whether these differences are also reflected in how people with chronic conditions differ in their experiences of co-care, with or without enabling technology. Therefore, this study aims to explore typical configurations of how the co-care system is experienced by individuals with chronic conditions who used an e-health service supporting self-monitoring and digital communication with primary care. We put forth the following research questions (RQs):

RQ 1: What profiles of experiences of the co-care system can be found among individuals with chronic conditions? How are these profiles characterized in terms of patients' age, gender, education level, type, and duration of condition?
RQ 2: How do self-rated health, self-efficacy in self-care, and satisfaction with healthcare differ between patients with different profiles at two time points?
RQ 3: How does profile membership shift before and after the introduction of an e-health service?
RQ 4: How does the perceived effectiveness of the e-health service differ between the co-care profiles?

## Methods

### Design and setting

This is a two-wave longitudinal questionnaire study set in a primary health care center in Sweden that pilot-tested an e-health service that involved shifting tasks and activities from primary care providers to patients. The e-health service included monitoring devices (activity tracker bracelet, blood pressure cuff, and scale) and a smartphone application. Self-measurements, trends, and alerts were available for both the individuals and their primary care practitioners, and they could communicate asynchronously through chat and video. More details about the e-health service and its use have been published previously (21, 22).

#### Recruitment

Participants were recruited among patients who participated in the primary care center's e-health service pilot. Individuals older than 18 and diagnosed with hypertension, chronic heart failure, or mental health conditions (e.g., stress-related ill-health, insomnia, anxiety, and depressive disorders) were eligible for the pilot. To be included in the pilot, they were also required to have a smartphone and email account and be able to communicate in Swedish (as the smartphone application was in Swedish). Eligible participants were identified by primary care staff and informed about the pilot via phone. They were then invited to a group enrollment session where they also received information and an invitation to participate in the questionnaire study. Informed consent was obtained electronically. The project followed the Helsinki Declaration guidelines, and it was approved by the Regional Ethical Review Board of Stockholm (reference numbers 2018/625-31/5 and 2018/1717-32).

### Data collection

Data were collected at two time points (November 2018 and June 2019) using a web-based questionnaire. The data collection at Time 1 (T1) took place directly after enrollment in the e-health pilot project (i.e., when the participants had just been introduced to the e-health service). The data collection at Time 2 (T2) took place 7 months later. Thus, the second assessment of co-care experiences involves e-health components, whereas the first measurement reflects a more traditional service delivery model with physical visits and occasional phone consultations. The collected data have been previously used and published as part of the psychometric evaluation of the Distribution of Co-Care Activities (DoCCA) scale (10). The evaluation supported a second-order model consisting of five subscales, indicated good-to-excellent reliability of the subscales (Cronbach's α values between 0.79 and 0.93), and satisfactory test–retest reliability. Further, validity of the instrument was supported by correlations with self-efficacy in self-care and satisfaction with healthcare in expected directions.

### Respondents

The T1 questionnaire was distributed to 308 patients. Reminders were sent after the first and second weeks, resulting in a response rate of 55%. The second questionnaire was distributed to the same 308 recipients with a response rate of 41% after two reminders. In all, 180 participants responded to at least one of the questionnaires and consented to participation; these patients make up the panel sample for this study. Of these, 117 responded to both questionnaires and 96 provided complete answers at both time points.

### Questionnaire

#### Co-care clustering variables

Co-care was measured with the five subscales of the 20-item DoCCA scale (10) to assess how patients perceive a chronic care management system consisting of both self-care and healthcare (with or without the support of e-health services). *Needs support* (four items) measures the extent to which an individual receives the support they need to take care of their health, regardless of the source of that support (e.g., “Do you feel that you get the help and support you need to take care of your health?”). *Goal orientation* (four items) measures the extent to which the co-care system is oriented toward goals that the individual values (e.g., “Do you feel healthcare supports you in achieving your goals?”). *Demands* (four items) measures cognitive and emotional demands associated with taking care of one's health (e.g., “When you take care of your health, do you feel that you need to keep track of many things at once?”). *Role clarity* (five items) measures the degree to which responsibilities and expectations are clear and are justified, as well as how the distribution of responsibility is perceived (e.g., “Do you feel that responsibility is reasonably distributed between you and healthcare?”). *Unnecessary tasks* (three items) measures tasks that do not need to be performed or could be better performed by someone else (e.g., “Do you feel that your self-care includes tasks that do not really make sense?”). Conceptually, the items concerning demands, role clarity, and unnecessary tasks form a dimension that reflects the way people perceive the activities involved in taking care of a chronic condition. However, the five subscales are analyzed separately. Items were rated on a 5-point response scale ranging from 1 to 5, with 1 indicating *to a very low degree* or *never/almost never* and 5 indicating *to a very high degree* or *always*. For items concerning demands and unnecessary tasks, a low value is positive, whereas a high value is positive for the other subscales.

#### Background and exogenous variables

Background variables, including age, gender, education level, and the type as well as duration of the health condition for which the e-health service was used were collected at T1 to describe the demographics of the latent profiles. Exogenous variables, including self-rated health, self-efficacy in self-care, and satisfaction with healthcare, were assessed at T1 and T2 to test for differences between latent profiles. At T2, we also assessed perceptions of the effectiveness of the e-health service.

Self-rated health was measured with two items. One assesses the general perception of one's health (“How would you rate your general health status?”), and the other assesses perceived general health compared to others of the same age (“How would you assess your general health status compared to that of others of your own age?”) (23). Items were rated on a 5-point response scale from 1 (*very bad*/*bad*) to 5 (*very good*/*excellent*).

Self-efficacy in self-care was measured with the self-efficacy scale in self-care (24), which includes six items measuring an individual's confidence in their ability to manage a condition (e.g., “How certain are you that you can affect your symptoms?”). Responses were given on a 4-point scale ranging from 1 (*very uncertain*) to 4 (*very certain*).

Satisfaction with healthcare was measured with two items adapted from the Swedish national patient survey: one assessing the satisfaction with overall care at the specific primary care center (“Overall, how do you value the care you have received at [name of primary care center] during the past 6 months?”) and the other assessing satisfaction with healthcare accessibility (“Do you feel that healthcare is accessible when you need it?”). Items were rated on a 5-point scale ranging from 1 (*bad* or *never/almost never*) to 5 (*excellent* or *always*).

Perceived effectiveness of e-health was measured with four items assessing the extent to which the e-health service was perceived to have influenced quality of care, participation in care, collaboration with primary care, and communication with primary care (e.g., “How has the use of [e-health service] affected the quality of your care?”). Items were rated on a 5-point response scale, ranging from −2 (*significantly deteriorated*) to 2 (*significantly improved*).

### Analytical strategy

Analyses were performed in R (25). Latent factor scores from a confirmatory factor analysis with full information maximum likelihood (FIML) for co-care variables and the exogenous variables were computed using the lavaan R package (26) and were used as input variables in the latent profile analysis (LPA). We used the R package tidyLPA (27) to perform the LPA. In our specification, each variable was allowed to have a different amount of variation in each profile, making it more realistic compared to the fixed variances specification (28). To make the model less computationally intensive given the relatively low sample size, covariances were constrained to 0 (i.e., the variables were not allowed to covary over and above their association as part of the same profile). This specification corresponds to Model 2 in tidyLPA (varying variances, covariances fixed to 0) or mclust Model VVI (29).

Models with one to 10 profiles were tested to identify the optimal number of profiles. We used several criteria to determine the optimal number of profiles (30): Akaike's information criterion (AIC), Bayesian information criterion (BIC), the sample-size adjusted BIC (SABIC), and the bootstrapped likelihood ratio test (BLRT). A better model fit is indicated by lower AIC, BIC, and SABIC values, as well as a statistically significant BLRT test, indicating that the target profile solution (*n*) fits better than a solution with one fewer profile (*n*−1) does. We also examined the entropy criterion, which indicates how accurately people are categorized into their respective profiles. Its value ranges from 0 to 1, and higher entropy values indicate a better fit for a given solution (31). We sought an entropy above 0.80, even though no definitive conventional cutoff criterion exists (32). Although the entropy criterion is considered a useful tool to assess classification accuracy (33, 34), it should not be used to determine the optimal number of profiles (34, 35). In addition to the fit criteria, we considered meaningfulness and parsimony of the latent profiles to choose the optimal solution, as well as the size of the smallest profile (28, 36).

This profile enumeration process was done separately at the time point for each wave because the number and nature of the profiles might differ between waves (37). To aid the interpretation of the results, *z* scores with a mean of 0 and standard deviation of 1 were used. Due to potentially uneven cluster sample sizes, a non-parametric Kruskall-Wallis test for multiple groups along with pairwise comparisons with Bonferroni correction for multiple comparisons were performed to examine differences between the latent profiles concerning exogenous factors. The dunn.test package in R (38) was used for pairwise comparisons; psych (39) was used for calculating descriptive statistics of raw scores and exogenous variables (means and variances); ggplot2 (40) was used to produce profile plots; and ggsankey (41) was used to produce a Sankey diagram illustrating profile transitions.

## Results

### Profile solutions

Models specifying an increasing number of clusters were examined to determine the best-fitting model for the data at T1 and T2, respectively, based on latent factor scores from of the five DoCCA subscales.

#### Best-fitting profile solution at T1

Table 1 shows that the model fit improved for each added profile, whereas adding a fifth profile did not significantly improve model fit (non-significant BLRT). Among the four solutions specifying one through four profiles, the model with four profiles had the best fit indices (the lowest AIC, BIC, and SABIC), good entropy (0.93), and acceptable size of the smallest cluster (*n* = 25, 14%). Based on these parameters, a four-cluster solution was chosen.

**Table 1:** Model fit indices from the latent profile analyses of co-care experiences at T1 (*N* = 180).

**Profiles**	**LogLik**	**AIC**	**BIC**	**SABIC**	**Entropy**	***n* min**	**BLRT (val)**	**BLRT (*p)***
1	−1,274.54	2,569.08	2,601.00	2,569.33	1	1	–	–
2	−1,135.31	2,312.63	2,379.68	2,313.17	0.86	0.42	278.45	0.01
3	−1,034.39	2,132.78	2,234.95	2,133.61	0.9	0.18	201.85	0.01
**4**	**−961.83**	**2,009.66**	**2,146.95**	**2,010.77**	**0.93**	**0.14**	**145.12**	**0.01**
5	−945.69	1,999.38	2,171.8	2,000.78	0.94	0.03	32.28	0.14
6	−894.95	1,919.89	2,127.43	1,921.58	0.93	0.03	101.49	0.01
7	−869.11	1,890.22	2,132.88	1,892.19	0.94	0.04	51.68	0.01
8	−837.35	1,848.71	2,126.5	1,850.97	0.94	0.05	63.51	0.01
9	−827.15	1,850.3	2,163.21	1,852.84	0.93	0.04	20.41	0.35
10	−824.48	1,866.97	2,215.00	1,869.8	0.94	0.04	5.33	0.91

LogLik, loglikelihood; AIC, Akaike Information Criterion; BIC, Bayesian Information Criterion; SABIC, sample-size Adjusted BIC; n min, Proportion of the sample assigned to the smallest class (based on most likely class membership); BLRT (val), Bootstrap Likelihood Ratio Test value; BLRT (p), p-value for the Bootstrap Likelihood Ratio Test. Bold indicates the profile solution, which is the final model.

#### Best-fitting profile solution at T2

Table 2 shows that the model fit improved for each added profile, with three large drops in AIC and SABIC: between two and three profiles, between four and five profiles, and between five and six profiles. We chose a five-profile solution due to meaningful differentiation between profiles with regards to the variables that make up the profiles and because additional profiles (above five) did not add qualitatively new profiles. The chosen solution had good entropy (0.93), though the size of the smallest cluster was barely acceptable (*n* = 7, 4%).

**Table 2:** Model fit indices from the latent profile analyses of co-care experiences at T2 (*N* = 180).

**Profiles**	**LogLik**	**AIC**	**BIC**	**SABIC**	**Entropy**	***n* min**	**BLRT (val)**	**BLRT (*p*)**
1	−1,274.54	2,569.08	2,601	2,569.33	1	1	–	–
2	−1,114.77	2,271.55	2,338.6	2,272.09	0.88	0.32	319.53	0.01
3	−1,002.01	2,068.02	2,170.2	2,068.85	0.9	0.27	225.52	0.01
4	−979.63	2,045.26	2,182.55	2,046.37	0.92	0.04	44.77	0.06
**5**	**−916.58**	**1,941.16**	**2,113.58**	**1,942.56**	**0.93**	**0.04**	**126.09**	**0.01**
6	−857.67	1,845.34	2,052.88	1,847.03	0.93	0.05	117.82	0.01
7	−836.06	1,824.13	2,066.79	1,826.1	0.93	0.05	43.21	0.02
8	−816.4	1,806.8	2,084.59	1,809.06	0.95	0.03	39.32	0.01
9	−822.67	1,841.35	2,154.26	1,843.89	0.95	0.03	−12.55	0.96
10	−780.3	1,778.61	2,126.64	1,781.44	0.95	0.03	84.74	0.01

LogLik, loglikelihood; AIC, Akaike Information Criterion; BIC, Bayesian Information Criterion; SABIC, sample-size Adjusted BIC; n min, Proportion of the sample assigned to the smallest class (based on most likely class membership); BLRT (val), Bootstrap Likelihood Ratio Test value; BLRT (p), p-value for the Bootstrap Likelihood Ratio Test. Bold indicates the profile solution, which is the final model.

### Typical configurations of co-care experiences (RQ 1)

#### Profile configurations at T1

**Figure 1:**
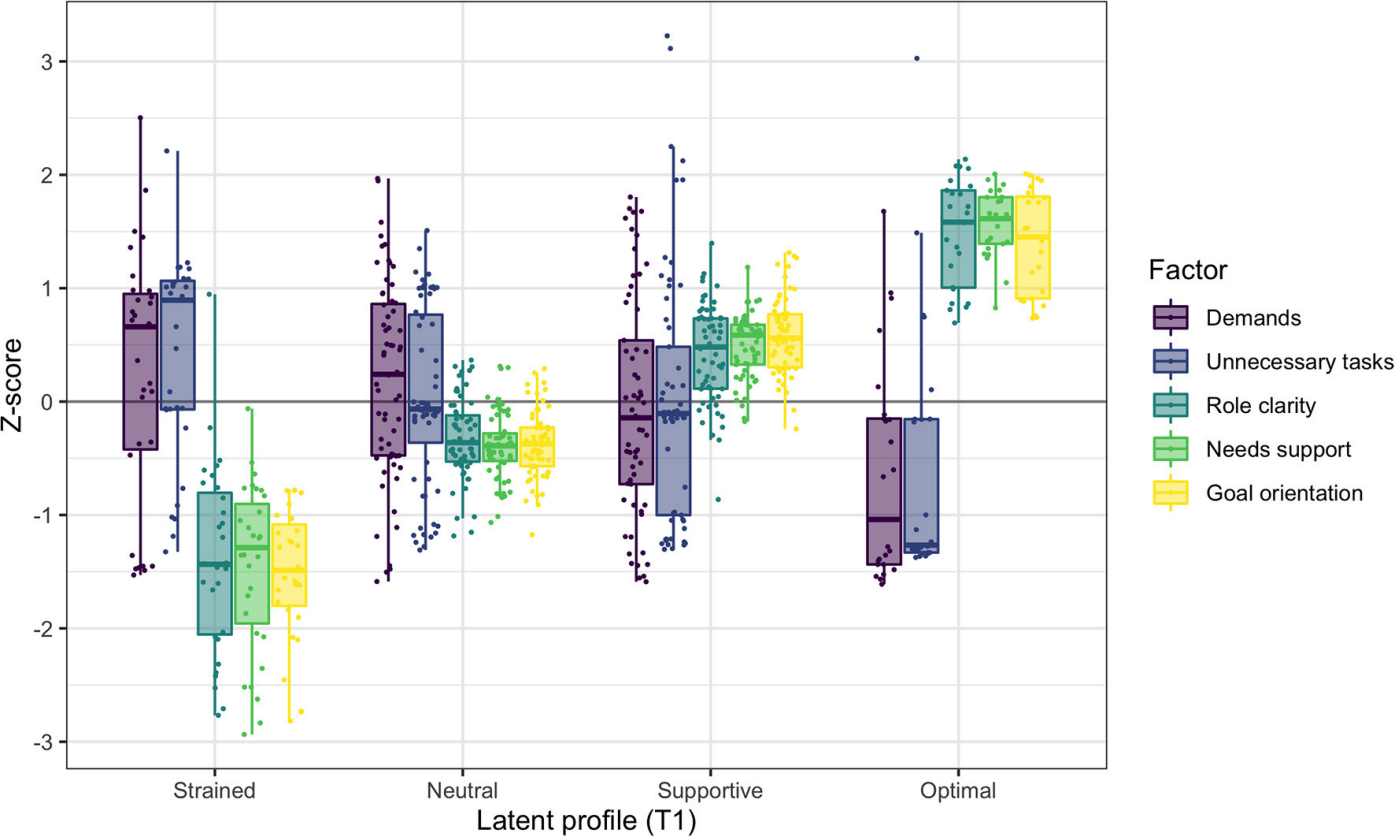
Co-care profiles at T1, illustrated with boxplots and individual data points of the co-care factor *z* scores (*M* = 0, *SD* = 1).

The configurations of the four profiles in the model at T1 are presented in Figure 1, with means and variances of the five co-care factors in Table 3. Background information is presented in Table 4.

**Table 3:** Means and variances of the raw scores and *z* scores of the five co-care factors at T1 (*N* = 180).

	**Latent profile**											
	Strained (*n =* 31*)			Neutral (*n =* 63*)			Supportive (*n =* 61*)			Optimal (*n =* 25*)		
	***n***	**Mean**	**Variance**	***n***	**Mean**	**Variance**	***n***	**Mean**	**Variance**	***n***	**Mean**	**Variance**
**Raw scores**												
Demands	30	2.73	1.32	56	2.7	0.88	58	2.47	1.02	25	1.87	0.94
Unnecessary tasks	30	2.73	0.58	55	2.25	0.42	58	2.16	0.86	24	1.61	0.81
Role clarity	30	2.33	0.59	56	3.16	0.12	58	3.71	0.2	24	4.53	0.22
Needs support	30	2.23	0.49	56	3.11	0.1	58	3.84	0.07	24	4.78	0.08
Goal orientation	30	2.16	0.35	56	3.08	0.13	58	3.9	0.11	24	4.52	0.24
	**Latent profile**							
	**Strained (*****n** =* **31)**		**Neutral (*****n** =* **63)**		**Supportive (*****n** =* **61)**		**Optimal (*****n** =* **25)**	
	**Mean**	**Variance**	**Mean**	**Variance**	**Mean**	**Variance**	**Mean**	**Variance**
***Z*** **scores**								
Demands	0.21	1.25	0.23	0.73	−0.07	0.93	−0.67	0.83
Unnecessary tasks	0.41	0.78	0.05	0.62	−0.03	1.18	−0.59	1.21
Role clarity	−1.36	0.66	−0.32	0.13	0.44	0.19	1.5	0.21
Needs support	−1.42	0.54	−0.38	0.09	0.51	0.08	1.55	0.09
Goal orientation	−1.49	0.36	−0.36	0.09	0.59	0.11	1.42	0.2

FIML was used to estimate means and variances for z scores, accounting for missing data. *The n indicates the size of the profile; due to missingness in the raw data, the actual n for each factor is provided for the raw scores. For the z scores, the n for each factor is equal to the size of the profile.

**Table 4:** Background variables of the profiles at T1 (*N* = 170^a^).

	**Latent profile**			
**Characteristic**	**Strained (*n* = 31)**	**Neutral (*n* = 63)**	**Supportive (*n* = 61)**	**Optimal (*n* = 25)**
**Age, mean (SD)**	54.73 (13.95)	56.42 (14.98)	60.05 (10.85)	61.80 (11.09)
**Gender**, ***n*** **(%)**				
Female	15 (50%)	35 (61%)	28 (48%)	19 (76%)
Male	15 (50%)	22 (39%)	30 (52%)	6 (24%)
**Education**, ***n*** **(%)**				
Elementary school	1 (3.3%)	4 (7.0%)	6 (10%)	1 (4.0%)
High school	13 (43%)	21 (37%)	27 (47%)	10 (40%)
Higher education	16 (53%)	32 (56%)	24 (41%)	14 (56%)
No completed education	0 (0%)	0 (0%)	1 (1.7%)	0 (0%)
**Chronic condition**^**b**^, ***n*** **(%)**				
Hypertension	14 (47%)	37 (65%)	43 (74%)	15 (60%)
Heart failure	3 (10%)	3 (5.3%)	5 (8.6%)	1 (4.0%)
Mental illness	8 (27%)	12 (21%)	7 (12%)	7 (28%)
Other	5 (17%)	13 (23%)	8 (14%)	4 (16%)
Uncertain	6 (20%)	1 (1.8%)	3 (5.2%)	0 (0%)
**Duration of chronic condition**, ***n*** **(%)**				
≤ 1 year	7 (23%)	8 (14%)	7 (12%)	8 (32%)
>1 and ≤ 3 years	6 (20%)	11 (19%)	19 (33%)	1 (4.0%)
>3 and ≤ 5 years	0 (0%)	12 (21%)	9 (16%)	4 (16%)
>5 and ≤ 10 years	10 (33%)	10 (18%)	8 (14%)	7 (28%)
>10 years	6 (20%)	15 (26%)	14 (24%)	4 (16%)
N/A	1 (3.3%)	1 (1.8%)	1 (1.7%)	1 (4.0%)
**(Missing)**^**a**^, ***n***	1	6	3	0

^a^All background variables were collected at T1 and are missing for 10 individuals in the study population who did not respond to the T1 questionnaire; ^b^The condition for which the e-health service is used. More than one condition possible.

**Profile 1 at T1: Strained (*n* =** **31, 17%):** Individuals in the strained profile had the least beneficial combinations of co-care experiences at T1, characterized by experiences of unclear roles, low needs support, and poor goal orientation. Most members of this profile also perceived being exposed to unnecessary tasks and demands, although variation was observed as positive and negative reports of these two factors. Half of the profile members were female, with a median age of 55 years. Almost half (47%) used the e-health service for high blood pressure and 27% for mental illness. Around half (53%) had known about their chronic condition for more than 5 years, whereof 20% had known about it for more than 10 years. Notably, 20% did not know for which condition they used the e-health service.

**Profile 2 at T1: Neutral (*n* =** **63, 35%)**. The neutral profile was close to the mean of the sample on all clustering variables. However, although each variable was close to the sample mean, they were all slightly shifted toward the negative side: slightly more demands and unnecessary tasks, and slightly less needs support, poorer goal orientation, and more unclear roles. Similar to the strained profile, reports of perceived demands and unnecessary tasks varied substantially, covering positive as well as negative experiences. This profile had a higher ratio of female members (61%), with a median age of 56 years. Almost two-thirds (65%) used the e-health service for high blood pressure, whereas 23% used it for other conditions and 21% for mental illness. One-fourth (26%) had been diagnosed more than 10 years ago; the time since diagnosis for other members was quite evenly distributed, ranging from < 1 year to 10 years.

**Profile 3 at T1: Supportive (*n* =** **61, 34%):** The supportive profile was characterized by high goal orientation, needs support, and role clarity. The experiences of demands and unnecessary tasks varied substantially and averaged close to the sample mean. Thus, the profile included individuals experiencing high as well as low demands and unnecessary tasks. Nearly half (48%) of the profile members were female, with a mean age of 60 years, thus somewhat older than the strained and neutral profiles. The majority (74%) used the e-health service for high blood pressure. One-third (33%) had known about their chronic condition for 1 to 3 years and one-fourth (24%) had been diagnosed more than 10 years ago.

**Profile 4 at T1: Optimal (*n* =** **25, 14%):** The optimal profile has the most positive configurations of co-care experiences, characterized by experiences of higher needs support, goal orientation, and clearer roles compared to other profiles. Further, most members experienced fewer demands and unnecessary tasks than members of the other profiles did. The optimal profile had the highest ratio of female members (76%) and the highest mean age (62 years). The majority (60%) used the e-health service for high blood pressure and 28% used it for mental illness. One-third (32%) had been diagnosed within the past year and 28% had known about their chronic condition for 5 to 10 years.

#### Profile configurations at T2

**Figure 2:**
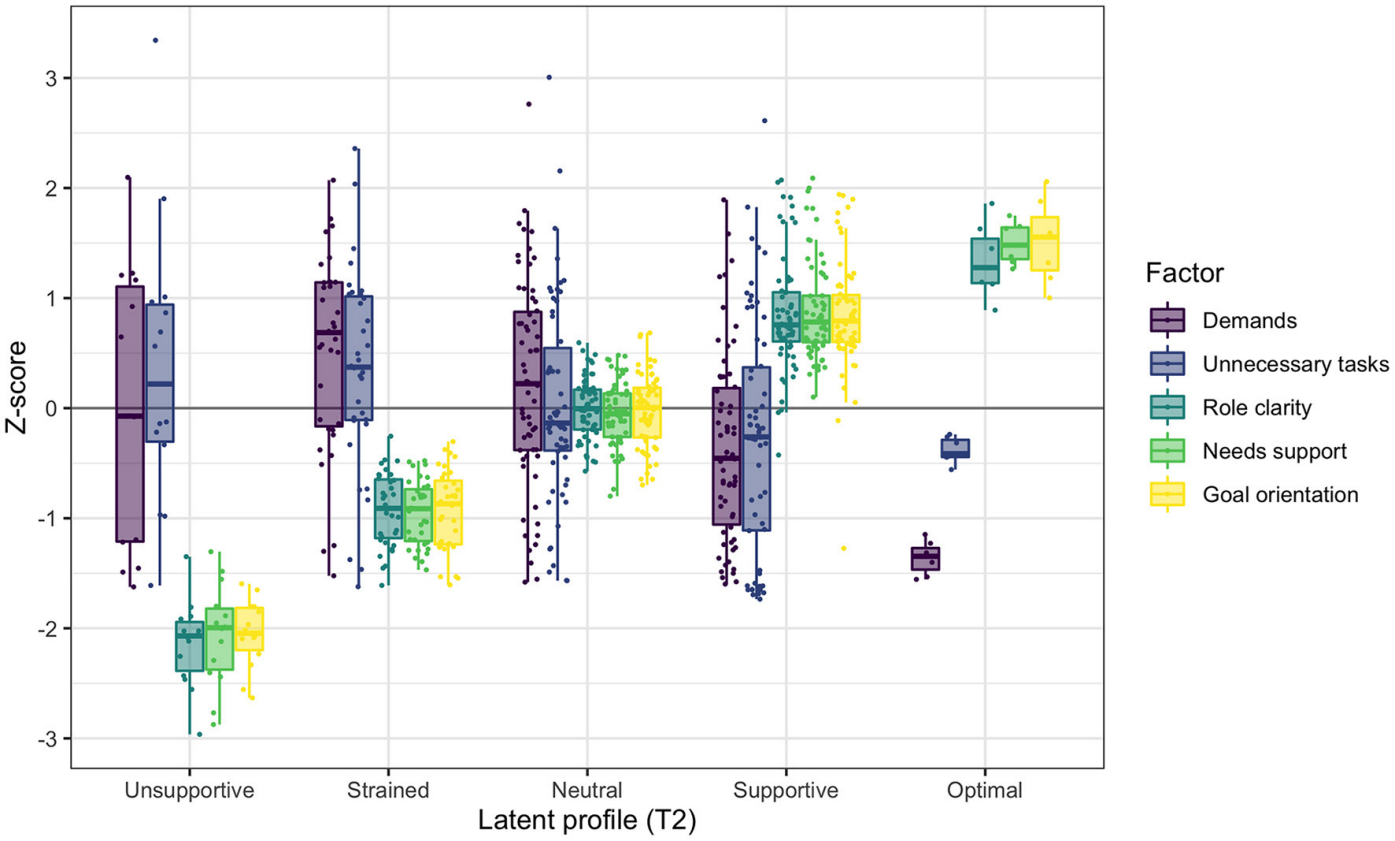
Co-care profiles at T2, illustrated with boxplots and individual data points of the co-care factor *z* scores (*M* = 0, *SD* = 1).

The configurations of the five profiles in the model at T2 are presented in Figure 2, with means and variances of the five co-care factors in Table 5. When labeling the T2 profiles, we retained the T1 labels to reflect similarity in patterns of the co-care factors, although levels may differ. Background information is presented in Table 6.

**Table 5:** Means and variances of the raw scores and *z* scores of the five co-care factors at T2 (*N* = 180).

	**Latent profile**														
	Unsupportive (*n =* 14*)			Strained (*n =* 35*)			Neutral (*n =* 62*)			Supportive (*n =* 62*)			Optimal (*n =* 7*)		
	***n***	**Mean**	**Variance**	***n***	**Mean**	**Variance**	***n***	**Mean**	**Variance**	***n***	**Mean**	**Variance**	***n***	**Mean**	**Variance**
**Raw scores**															
Demands	9	2.25	1.46	21	2.75	0.71	43	2.56	0.72	48	1.95	0.59	5	1.25	0.1
Unnecessary tasks	9	2.48	1.04	21	2.48	0.79	42	2.37	0.56	48	2	0.76	4	2	0.07
Role clarity	9	1.76	0.25	21	2.62	0.1	43	3.33	0.09	48	4.07	0.24	5	4.4	0.16
Needs support	10	1.73	0.34	21	2.75	0.08	43	3.32	0.12	48	4.19	0.19	5	4.75	0.06
Goal orientation	9	1.69	0.2	21	2.62	0.15	43	3.4	0.13	48	4.06	0.29	5	4.65	0.12
	**Latent profile**									
	**Unsupportive (*****n** =* **14)**		**Strained (*****n** =* **35)**		**Neutral (*****n** =* **62)**		**Supportive (*****n** =* **62)**		**Optimal (*****n** =* **7)**	
	**Mean**	**Variance**	**Mean**	**Variance**	**Mean**	**Variance**	**Mean**	**Variance**	**Mean**	**Variance**
***Z*** **scores**										
Demands	0.05	1.4	0.49	0.78	0.23	0.89	−0.37	0.71	−1.36	0.02
Unnecessary tasks	0.37	1.54	0.32	0.81	0.06	0.81	−0.28	1.09	−0.38	0.01
Role clarity	−2.16	0.13	−0.91	0.11	0.01	0.07	0.85	0.28	1.34	0.09
Needs support	−2.07	0.2	−0.95	0.09	−0.04	0.09	0.88	0.21	1.5	0.03
Goal orientation	−2.06	0.09	−0.93	0.14	−0.02	0.11	0.84	0.27	1.52	0.12

FIML was used to estimate means and variances for z scores, accounting for missing data. *The n indicates the size of the profile; due to missingness in the raw data, the actual n for each factor is provided for the raw scores. For the z scores, the n for each factor is equal to the size of the profile.

**Table 6:** Background variables of the profiles at T2 (*N* = 170^a^).

	**Latent profile**				
	**Unsupportive (*n =* 14)**	**Strained (*n =* 35)**	**Neutral (*n =* 62)**	**Supportive (*n =* 62)**	**Optimal (*n =* 7)**
**Age, mean (SD)**	56.00 (12.91)	55.79 (14.88)	56.18 (14.61)	60.77 (10.40)	67.17 (5.34)
**Gender**, ***n*** **(%)**					
Female	8 (62%)	21 (64%)	31 (55%)	32 (52%)	5 (83%)
Male	5 (38%)	12 (36%)	25 (45%)	30 (48%)	1 (17%)
**Education**, ***n*** **(%)**					
Elementary school	0 (0%)	4 (12%)	4 (7.1%)	3 (4.8%)	1 (17%)
High school	5 (38%)	14 (42%)	19 (34%)	30 (48%)	3 (50%)
Higher education	8 (62%)	15 (45%)	32 (57%)	29 (47%)	2 (33%)
No completed education	0 (0%)	0 (0%)	1 (1.8%)	0 (0%)	0 (0%)
**Chronic condition**^**b**^, ***n*** **(%)**					
Hypertension	7 (54%)	19 (58%)	32 (57%)	47 (76%)	4 (67%)
Heart failure	0 (0%)	2 (6.1%)	2 (3.6%)	8 (13%)	0 (0%)
Mental illness	1 (7.7%)	9 (27%)	14 (25%)	9 (15%)	1 (17%)
Other	2 (15%)	6 (18%)	14 (25%)	7 (11%)	1 (17%)
Uncertain	5 (38%)	1 (3.0%)	3 (5.4%)	1 (1.6%)	0 (0%)
**Duration of chronic condition**, ***n*** **(%)**					
≤ 1 year	6 (46%)	4 (12%)	9 (16%)	10 (16%)	1 (17%)
>1 and ≤ 3 years	1 (7.7%)	8 (24%)	16 (29%)	10 (16%)	2 (33%)
>3 and ≤ 5 years	1 (7.7%)	3 (9.1%)	8 (14%)	13 (21%)	0 (0%)
>5 and ≤ 10 years	2 (15%)	9 (27%)	11 (20%)	11 (18%)	2 (33%)
>10 years	2 (15%)	9 (27%)	10 (18%)	18 (29%)	0 (0%)
N/A	1 (7.7%)	0 (0%)	2 (3.6%)	0 (0%)	1 (17%)
**(Missing)**^**a**^, ***n***	1	2	6	0	1

^a^All background variables were collected at T1 and are missing for 10 individuals in the study population who did not respond to the T1 questionnaire; ^b^The condition for which the e-health service is used. More than one condition possible.

**Profile 1 at T2: Unsupportive (*n* =** **14, 8%):** Individuals in the unsupportive profile had the least beneficial combination of co-care experiences at T2. Inspection of raw scores indicates that this profile was characterized by lower ratings of needs support, poorer goal orientation, and less role clarity compared to the strained profile at T1. The experiences of demands and unnecessary tasks varied, averaging slightly above the population mean. Most members in this profile were female (62%) and the mean age was 56 years. At the initiation of the pilot (T1), 54% reported that they used the e-health service for high blood pressure management, although 38% reported that they did not know the condition for which they were using the service. Almost half (46%) had been newly diagnosed, within a year prior to the pilot.

**Profile 2 at T2: Strained (*n* =** **35, 19%):** The strained profile at T2 was characterized by low role clarity, needs support, and goal orientation, but higher than was the case in the strained profile at T1, as indicated by a qualitative assessment of raw scores (Tables 3, 5). The experiences of demands and unnecessary tasks varied, also here slightly above the population mean. Sixty-four percent were female and the mean age was 56 years, similar to the demographics of the unsupportive profile. In this profile, 27% used the e-health service for mental health conditions, which was a higher proportion than observed in any other profile at T2. However, the majority (58%) used the e-health service for high blood pressure; 54% had been diagnosed more than 5 years prior to the pilot, whereof half were diagnosed more than 10 years prior.

**Profile 3 at T2: Neutral (*n* =** **62, 34%):** The neutral profile at T2 shares similarities with the neutral profile at T1, characterized by neutral experiences with average *z* scores for all co-care factors centering on the mean of the study population. As in other profiles, the variability of experienced demands and unnecessary tasks was high. Further, reports of role clarity, goal orientation, and needs support were on average slightly higher than they were in the neutral profile at T1 (Tables 3, 5). Slightly more than half (55%) of the profile's members were female and the mean age was 56 years. In this profile, 57% used the e-health service for high blood pressure, 25% for mental illness, and 25% for other conditions. The time since diagnosis prior to engaging in the pilot was quite evenly distributed, ranging from < 1 to more than 10 years, with the largest proportion of individuals (29%) having known about their chronic condition for 1 to 3 years.

**Profile 4 at T2: Supportive (*n* =** **62, 34%):** The supportive profile at T2 was characterized by positive experiences of resources in terms of role clarity, needs support, and goal orientation, slightly higher than observed for the supportive profile at T1. Similar to the other profiles, the demands and unnecessary tasks varied, averaging slightly below the mean of the study population and with raw scores indicating lower demands in the supportive profile at T2 than occurred at T1. Around half of the profile members were female (52%) with a mean age of 61 years. The majority (76%) used the e-health service for high blood pressure, and 13% had heart failure, which was less common or not at all prevalent in other profiles. In addition, this profile had the highest proportion (29%) of members who had been diagnosed more than 10 years prior to the pilot.

**Profile 5 at T2: Optimal (*n* =** **7, 4%):** The optimal profile at T2 was the smallest profile and the one with the most beneficial combination of co-care factors at T2. Particularly, all members in this profile experienced fewer demands and unnecessary tasks than the sample mean. Further, all members had positive experiences of role clarity, needs support, and goal orientation, which were higher than observed in all other profiles. Compared to the optimal profile at T1, inspection of raw scores indicates that the optimal profile at T2 was characterized by fewer demands but more unnecessary tasks. The majority (83%) of profile members were female, with a higher mean age (67 years) than all other profiles had. Around two-thirds (67%) used the e-health service for high blood pressure, whereas 17% used it for mental health conditions and 17% for other conditions. Half of the members had been diagnosed within 3 years prior to the pilot, whereof most were diagnosed more than 1 year prior, and 33% had been diagnosed between 5 and 10 years prior.

### Profile comparisons based on self-rated health, self-efficacy in self-care, and satisfaction with healthcare (RQ 2)

#### Profile comparisons at T1

The analysis of exogenous variables at T1 (Table 7) demonstrates that the profiles could be distinguished based on self-rated health, self-efficacy in self-care, and levels of satisfaction with healthcare (Table 8). The supportive and optimal profiles indicated high self-rated health and self-efficacy in self-care, and they differed significantly from the strained and the neutral profiles. However, there were no differences between the strained and neutral profiles, or between the supportive and optimal profiles. The satisfaction with healthcare was the highest in the optimal profile, followed by the supportive profile; these profiles differed significantly from all others, whereas the difference between the strained and neutral profiles was marginal (*p* = 0.067).

**Table 7:** Means and variances of the raw scores and *z* scores of exogenous variables at T1 (*N* = 180).

	**Latent profile**											
	Strained (*n =* 31*)			Neutral (*n =* 63*)			Supportive (*n =* 61*)			Optimal (*n =* 25*)		
	***n***	**Mean**	**Variance**	***n***	**Mean**	**Variance**	***n***	**Mean**	**Variance**	***n***	**Mean**	**Variance**
**Raw scores**												
Self-rated health	30	3.06	0.96	57	3.24	0.69	58	3.67	0.55	25	3.71	0.71
Self-efficacy in self-care	30	2.69	0.34	56	2.79	0.24	58	2.96	0.27	25	3.33	0.22
Satisfaction with healthcare	30	3.16	0.76	57	3.63	0.31	58	4.29	0.26	25	4.6	0.19
	**Latent profile**							
	**Strained (*****n** =* **31)**		**Neutral (*****n** =* **63)**		**Supportive (*****n** =* **61)**		**Optimal (*****n** =* **25)**	
	**Mean**	**Variance**	**Mean**	**Variance**	**Mean**	**Variance**	**Mean**	**Variance**
***Z*** **scores**								
Self-rated health	−0.18	0.18	−0.09	0.12	0.07	0.14	0.29	0.1
Self-efficacy in self-care	−0.24	0.72	−0.21	0.48	0.21	0.46	0.31	0.53
Satisfaction with healthcare	−0.59	0.19	−0.22	0.1	0.27	0.1	0.62	0.06

FIML was used to estimate means and variances for z scores, accounting for missing data. *The n indicates the size of the profile; due to missingness in the raw data, the actual n for each factor is provided for the raw scores. For the z scores, the n for each factor is equal to the size of the profile.

**Table 8:** Pairwise comparisons of exogenous variables between latent profiles at T1 (*N* = 180).

	**Exogenous variable**					
	**Self-rated health**		**Self-efficacy in self-care**		**Satisfaction with healthcare**	
**Pairwise comparison**	***Z* statistic**	**Adjusted *p*-value**	***Z* statistic**	**Adjusted *p*-value**	***Z* statistic**	**Adjusted *p*-value**
Strained - neutral	−0.14	1	−0.91	1	−2.54	0.067
Strained - supportive	−2.8	**0.031**	−3.25	**0.007**	−7.32	**< 0.001**
Neutral - supportive	−3.27	**0.007**	−2.88	**0.024**	−5.89	**< 0.001**
Strained - optimal	−2.71	**0.04**	−4.77	**< 0.001**	−8.62	**< 0.001**
Neutral - optimal	−2.96	**0.018**	−4.59	**< 0.001**	−7.45	**< 0.001**
Supportive - optimal	−0.48	1	−2.39	0.102	−2.96	**0.018**
**Omnibus Kruskall-Wallis**	**chi-squared (** ***df*** **)**	***p*** **-value**	**chi-squared (** ***df*** **)**	***p*** **-value**	**chi-squared (** ***df*** **)**	***p*** **-value**
	18.04 (3)	< 0.001	31.64 (3)	< 0.001	109.08 (3)	< 0.001

Bold values indicate statistically significant differences between profiles (*p* < 0.05).

#### Profile comparisons at T2

The analysis of exogenous variables at T2 (Table 9) demonstrates that the profiles could be distinguished based on self-rated health, self-efficacy in self-care, and levels of satisfaction with healthcare (Table 10). Self-rated health was significantly higher in the optimal profile compared to all other profiles. The supportive and strained profiles also differed, with the supportive profile reporting higher self-rated health. The optimal and supportive profiles also reported higher self-efficacy in self-care and higher satisfaction with healthcare.

**Table 9:** Means and variances of the raw scores and *z* scores of exogenous variables at T2 (*N* = 180).

	**Latent profile**														
	Unsupportive (*n =* 14*)			Strained (*n =* 35*)			Neutral (*n =* 62*)			Supportive (*n =* 62*)			Optimal (*n =*7*)		
	***n***	**Mean**	**Variance**	***n***	**Mean**	**Variance**	***n***	**Mean**	**Variance**	***n***	**Mean**	**Variance**	***n***	**Mean**	**Variance**
**Raw scores**															
Self-rated health	10	3.07	1.12	21	2.92	0.64	43	3.34	0.92	48	3.55	0.58	5	4.53	0.03
Self-efficacy in self-care	10	2.55	1.02	21	2.52	0.15	43	2.82	0.26	48	3.05	0.21	5	3.5	0.15
Satisfaction with healthcare	9	2.74	0.64	21	3.27	0.42	42	3.91	0.23	47	4.33	0.23	5	4.93	0.02
Perceived impact of e-health	9	0.01	0.79	21	0.42	0.42	42	0.47	0.24	48	0.75	0.3	5	1.3	0.94
	**Latent profile**									
	**Unsupportive (*****n** =* **14)**		**Strained (*****n** =* **35)**		**Neutral (*****n** =* **62)**		**Supportive (*****n** =* **62)**		**Optimal (*****n** =* **7)**	
	**Mean**	**Variance**	**Mean**	**Variance**	**Mean**	**Variance**	**Mean**	**Variance**	**Mean**	**Variance**
***Z*** **scores**										
Self-rated health	−0.28	0.53	−0.24	0.08	−0.03	0.15	0.17	0.12	0.59	0.08
Self-efficacy in self-care	−0.15	0.76	−0.37	0.34	−0.04	0.55	0.16	0.35	1.16	0.04
Satisfaction with healthcare	−0.91	0.07	−0.56	0.1	0.02	0.12	0.41	0.1	0.85	0.05
Perceived impact of e-health	−0.46	0.29	−0.19	0.2	−0.05	0.14	0.2	0.18	0.54	0.5

FIML was used to estimate means and variances for z scores, accounting for missing data. *The n indicates the size of the profile; due to missingness in the raw data, the actual n for each factor is provided for the raw scores. For the z scores, the n for each factor is equal to the size of the profile.

**Table 10:** Pairwise comparisons of exogenous variables between latent profiles at T2 (*N* = 180).

	**Exogenous variable**							
	**Self-rated health**		**Self-efficacy in self-care**		**Satisfaction with healthcare**		**Perceived impact of e-health**	
**Pairwise comparison**	***Z* statistic**	**Adjusted *p*-value**	***Z* statistic**	**Adjusted *p*-value**	***Z* statistic**	**Adjusted *p*-value**	***Z* statistic**	**Adjusted *p*-value**
Unsupportive - strained	1.12	1	0.55	1	−1.31	1	−1.57	1
Unsupportive - neutral	−0.42	1	−1.43	1	−4.84	**< 0.001**	−3.17	**0.015**
Strained - neutral	−2.26	0.24	−2.84	**0.046**	−4.82	**< 0.001**	−2.08	0.376
Unsupportive - supportive	−1.53	1	−3.33	**0.009**	−7.44	**< 0.001**	−5.17	**< 0.001**
Strained - supportive	−3.81	**0.001**	−5.49	**< 0.001**	−8.46	**< 0.001**	−4.89	**< 0.001**
Neutral - supportive	−1.82	0.682	−3.12	**0.018**	−4.29	**< 0.001**	−3.31	**0.009**
Unsupportive - optimal	−3.63	**0.003**	−3.94	**0.001**	−6.38	**< 0.001**	−4.08	**< 0.001**
Strained - optimal	−4.91	**< 0.001**	−4.83	**< 0.001**	−6.13	**< 0.001**	−3.37	**0.008**
Neutral - optimal	−3.9	**0.001**	−3.51	**0.005**	−3.82	**0.001**	−2.39	0.168
Supportive - optimal	−3.08	**0.021**	−2.1	0.356	−1.88	0.594	−0.9	1
**Omnibus Kruskall-Wallis**	**chi-squared (df)**	***p*** **-value**	**chi-squared (df)**	***p*** **-value**	**chi-squared (df)**	***p*** **-value**	**chi-squared (df)**	***p*** **-value**
	30.87 (4)	< 0.001	46.6 (4)	< 0.001	117.95 (4)	< 0.001	46.02 (4)	< 0.001

Non-parametric tests due to large differences in group sizes; comparisons adjusted with Bonferroni correction for multiple comparisons. Bold values indicate statistically significant differences between profiles (*p* < 0.05).

### Shift in profile membership over time (RQ 3)

**Figure 3:**
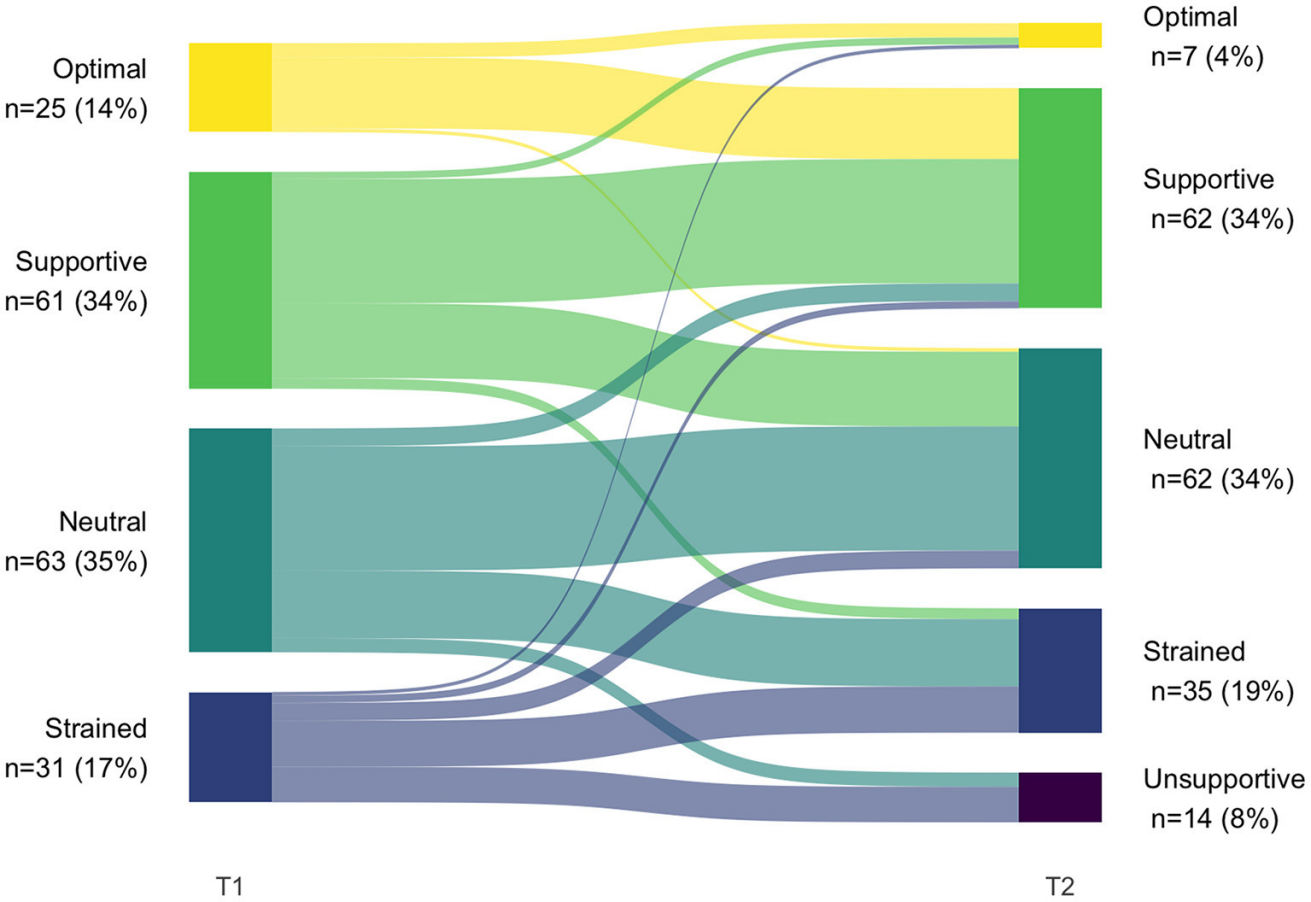
Pattern of latent profile transitions from T1 to T2 (*N* = 180).

Because different numbers of latent profiles were obtained at T1 and T2, there was no configural similarity. Therefore, we did not test for structural similarity (whether the profiles are characterized by similar levels on the profile indicators across groups) (37). The analysis of transitions between T1 and T2 was thus qualitative in nature (see Figure 3 and Table 11).

**Table 11:** Crosstabulation of latent profile transitions from T1 to T2 (*N* = 180).

	**T2 Latent profile**				
	**Unsupportive (*n =* 14)**	**Strained (*n =* 35)**	**Neutral (*n =* 62)**	**Supportive (*n =* 62)**	**Optimal (*n =* 7)**
**T1 Latent profile**	***n* (row %)**	***n* (row %)**	***n* (row %)**	***n* (row %)**	***n* (row %)**
Strained (*n* = 31)	10 (32%)	13 (42%)	5 (16%)	2 (6.5%)	1 (3.2%)
Neutral (*n* = 63)	4 (6.3%)	19 (30%)	35 (56%)	5 (7.9%)	0 (0%)
Supportive (*n* = 61)	0 (0%)	3 (4.9%)	21 (34%)	35 (57%)	2 (3.3%)
Optimal (*n* = 25)	0 (0%)	0 (0%)	1 (4.0%)	20 (80%)	4 (16%)

The most common transition (48% of patients, *n* = 87) was to the T2 equivalent of patients' T1 profile. Inspections of raw scores (Tables 3, 5) of the profiles that we deemed equivalent indicate a slight positive shift from T1 to T2, thus suggesting a potential improvement of co-care experiences. Eight percent (*n* = 15) shifted to a more positive co-care profile, whereas 43% (*n* = 78) shifted to a profile representing a more negative co-care experience.

Among those individuals who had better co-care experiences at T1 (i.e., supportive or optimal), the majority remained in one of these profiles at T2. However, more than one-third of the individuals in the supportive profile transitioned to a worse experience (neutral or strained). A similar pattern was seen for those in the neutral profiles, of whom more than one-third transitioned to the strained and unsupportive profiles. Finally, participants in the strained profile at T1 tended to retain negative experiences; 42% sustained the strained profile (albeit with a positive shift in raw scores) and 32% shifted to the unsupportive profile. However, about one-fourth transitioned to a more positive profile (ranging from neutral to optimal). Indeed, the strained profile at T1 was the only one in which people transitioned to the full range of profiles at T2, suggesting some individuals could experience major improvements in co-care experiences (shifting from strained at T1 to supportive or optimal at T2).

### Perceived effectiveness of the e-health service (RQ 4)

The members of the optimal and supportive profiles at T2 reported a more positive effect of the e-health service than those in the unsupportive and strained profiles did (Table 10). Inspection of raw scores (Table 9) reveals that none of the profiles indicated a negative effect on average, although the mean was around 0 for the unsupportive profile, indicating neither improvement nor worsening. The highest overall improvement was reported by members of the optimal profile, although the variance was large.

## Discussion

This study identified typical patterns in the ways a heterogeneous population of individuals with chronic conditions experienced co-care in a primary care setting. Our findings show that experiences of the co-care system can be summarized in distinct profiles ranging from unsupportive to optimal. The profiles differed primarily in terms of patients' perceived needs support, goal orientation, and role clarity. Further, they differed in patients' perceptions of self-rated health, self-efficacy in self-care, and satisfaction with healthcare, but not in profile members' genders, ages, educational levels, or types of chronic condition. The introduction of an e-health service was associated with different degrees of positive effects on the perceived quality of care, participation, collaboration, and communication with healthcare. However, both positive and negative shifts in the overall experiences of the co-care system were observed 7 months after the introduction of the e-health service.

From the perspective of a person living with a chronic condition, the task of taking care of one's health may be more manageable when one combines one's resources with resources offered by healthcare providers (or others) in a co-care system (9). The identified differences between profiles in their members' experiences of needs support and goal orientation provide justification for the importance of person-centered care (42), where it is central to orient care toward what matters for the person. In addition, the perception of role clarity was distinguished between the profiles, highlighting that a positive experience of the co-care system not only derives from interacting with healthcare professionals who are sensitive to one's needs and goals. Role clarity is the degree to which patients have a clear understanding of their as well as healthcare providers' responsibilities and mutual expectations and perceive that the responsibilities are adequately distributed (10). Role clarity reflects the patients' perceptions of the boundaries between self-care and healthcare. The possibility of sharing tasks and roles between actors in the co-care system as well as new ways of interacting with each other (e.g., through e-health services or new models of care) can also lead to increasingly blurred boundaries between self-care and healthcare (7). Research has shown that patients may lack clarity about their role in collaborative care models where healthcare professionals, patients, and families work together, which may be linked to insufficient guidance about how to distribute roles (19). This finding implies that when introducing new (e.g., digital) healthcare services or models of care that change the nature of collaborative work, healthcare professionals need to gain agreement with their patients about how to divide tasks and responsibilities, which should be addressed by organizational guidelines (43). The importance of such mutual agreement is strengthened by our findings that poor role clarity, needs support, and goal orientation were associated with poorer experiences of self-efficacy in self-care, satisfaction with care, and self-rated health.

Research has suggested that self-efficacy is an antecedent to self-care (2, 6, 44). Among patients with heart failure, which was one of the conditions addressed in this study, higher self-efficacy in self-care has been associated with better self-care performance and improved health outcomes (45, 46). Thus, in conceptualizing co-care as a system perspective of self-care, it is not surprising that patients in the profiles with more positive co-care experiences (i.e., the optimal and supportive profiles) also reported higher self-efficacy in self-care, self-rated health, and satisfaction with healthcare. However, our results do not reveal any causal relationships between the tested variables. Thus, it remains to be investigated whether self-efficacy in self-care, self-rated health, and satisfaction with healthcare can be influenced by improving the co-care experience (e.g., through e-health interventions), or whether patients who already rate these factors as high tend to experience co-care as more positive. A study that explored patients' use of an online patient portal found that patients with lower self-efficacy in self-care were more likely to use the portal, suggesting a greater need for interacting with a healthcare provider to manage their condition (47). This could imply that a co-care intervention using e-health may address the needs of patients experiencing low self-efficacy. Other factors (e.g., personal characteristics, health condition, or contextual factors) that can explain variations in self-efficacy in self-care, self-rated health, and satisfaction with healthcare should also be considered. For example, having multiple morbidities has been shown to contribute to lower self-efficacy in self-care, because multiple conditions make it more challenging to determine the cause of symptoms (48).

Patients' experiences of interactions with healthcare and e-health services can be expected to differ based on their sociodemographic characteristics and health condition (16, 20). Overall, we found members of different ages, genders, educational levels, as well as types and durations of chronic conditions in all profiles. This result suggests that differentiated co-care experiences may not easily be explained by individual factors, which reinforces previous observations that people's reactions to practices in healthcare (e.g., wearable technologies) can vary substantially, both between and within individuals (20). In one regard, this observation implies that optimal co-care experiences may be possible for all, but alternatively, it also means that co-care experiences are not easily predicted from variables that are readily available (e.g., sociodemographic variables or diagnostic data). More research on factors that might predict patients' experiences of co-care is needed.

Similar to the heterogeneity in patient characteristics, patients' perceptions of demands and unnecessary tasks varied substantially within profiles. Conceptually, demands and unnecessary tasks are two aspects that reflect how tasks are distributed in co-care (10). However, these factors revealed less pronounced differences between profiles than the other co-care factors did (i.e., role clarity, needs support, and goal orientation). One possible explanation may be that the perception of demands and unnecessary tasks when taking care of one's health may also reflect an individual's health status. Individuals with a high burden of illness may perceive higher demands and a greater number of unnecessary tasks than individuals who have a lower burden of illness, independent of how tasks are distributed, because having a chronic condition may be perceived as demanding and a cause of unnecessary tasks. Hence, because health conditions varied within profiles, we can also expect the perceptions of health-related demands and unnecessary tasks to vary. Complementing research that has shown that both good and poor health statuses can have a negative effect on people's self-management (49), our results indicate that the co-care experience can be positive (e.g., the supportive profile), even if the tasks involved in taking care of one's health are experienced as demanding or unnecessary. This outcome suggests that regardless of disease burden, patients may benefit from a co-care system that caters for productive interactions in health- and self-care.

The LPA resulted in different numbers of profiles at the two investigated time points, implying that latent profile transitions could only be evaluated qualitatively. Thus, due to the lack of configural and structural similarity, we could not fully answer RQ 3. However, our cautious conclusion is that the e-health service may have contributed to empowering some patients in their self-care because a slight majority of patients transitioned to a similar or more positive profile after the introduction of the e-health service. It should be considered that the introduction of e-health services might be a two-edged sword, contributing to improved autonomy and empowerment, but also leading to blurred boundaries between healthcare and self-care, as discussed above (43). Nevertheless, our conclusion is supported by the profile members' ratings of the degree to which they experienced the e-health service's contribution to improved quality of care, participation, and collaboration as well as communication with healthcare providers. None of the profiles indicated that the e-health service had contributed to any deterioration. Ranging from the unsupportive to the optimal profile, the perceived effectiveness of the e-health service gradually increased. The unsupportive profile was overrepresented with people who (at baseline) were uncertain about why they were using the e-health service, which may provide some explanation for the absence of positive effects, as experienced by patients. This again underlines the importance of mutual agreements on the purpose and nature of collaborative work and the role of the components of the co-care system when introducing a new e-health service.

### Methodological considerations

Researchers have increasingly used LPA to understand the patient experience better [e.g., (50–52)]. Person-centered approaches provide researchers additional means to detect relatively homogeneous subpopulations of participants presenting qualitatively and quantitatively distinct configurations on a set of indicators (33). Thus, there may be different ways that the facets of co-care experiences combine, forming subpopulations sharing similar characteristics that cannot be detected with variable-oriented methods. Moreover, employing this analysis allowed us to understand the relations between a specific subgroup with outcomes relevant for their self-care. For example, in a previous analysis using a variable-oriented approach (10), we showed that satisfaction with care (and self-efficacy in self-care) was positively associated with role clarity, needs support, and goal orientation, but it was negatively associated with demands and unnecessary tasks. Although the person-oriented approach applied in this study partly supports these findings, it also revealed that the demands and unnecessary tasks had more complex associations, as indicated by profiles where patients experienced high demands and unnecessary tasks and were still associated with higher satisfaction with healthcare (and higher self-efficacy), as long as needs support, goal orientation, and role clarity was sufficient (e.g., the supportive profile).

#### Limitations

Some limitations need to be addressed. One limitation of our study is that the sample was relatively small for an LPA, whereas it has been suggested that samples of more than 500 participants are more appropriate (53). Although our analysis did not create convergence problems, it is likely that sample size was a limitation for identifying small subpopulations (54). For example, there might be smaller subgroups that are distinguished more by demands and unnecessary tasks than was found in the current study. Nevertheless, identifying small clusters may be more relevant for theoretical than for practical purposes, whereas being able to distinguish the main subgroups may be sufficient.

Another limitation is that although our data were from two time points, we used the information for a transition analysis rather than separating the profiles from the exogenous variables. Thus, reversed relationships between profiles and exogenous variables cannot be excluded (e.g., a relationship where satisfaction with care affects co-care experiences rather than the other way around). In addition, because the data at T2 were best represented by five profiles rather than the four found at T1, configural differences between time points prevented statistical analysis of transitions and their predictors.

### Conclusions

Our findings show it is possible to identify distinct subgroups of patients with different co-care experiences that are associated with differences in perceptions of self-efficacy in self-care, satisfaction with healthcare, and self-rated health. The profiles were not characterized by gender, age, or type of chronic condition, implying these factors do not predict experiences in co-care or the persons who may benefit from self-care interventions that influence how individuals interact and collaborate. Rather, the subgroups differed in their experiences of role clarity, needs support, and goal orientation. Thus, we may conclude that self-care interventions that aspire to support co-care need to consider not only person-centeredness (i.e., needs support and goal orientation), but also to make the distribution of roles and responsibilities explicit (i.e., role clarity). For example, when introducing a new e-health service, the potential consequences in terms of changed interaction patterns between patients and healthcare professionals should be addressed.

## Data availability statement

The original dataset presented in this article is not readily available because the ethical permit does not allow for participant's informed consent to be publicly shared. Requests to access the dataset should be directed to Karolinska Institutet's Research Data Office (rdo@ki.se). Additionally, an anonymized dataset with latent factor scores and the R code used to run the analyses can be found in the Swedish National Data Service: https://doi.org/10.48723/kzja-5k21.

## Ethics statement

The studies involving human participants were reviewed and approved by Regional Ethical Review Board of Stockholm, Sweden. The patients/participants provided their written informed consent to participate in this study.

## Author contributions

CW, HH, KK, and UvTS did the study conception and acquisition of data. MR and CW analyzed the data. CW created visualizations. CW, MR, HH, KK, and UvTS participated in significant decisions in the analytic process. CW, UvTS, and MR drafted the first version of the manuscript. All authors contributed with critical insights during an iterative writing process, approved, and are fully accountable for the final version.

## Funding

This research was funded by a project grant from the Kamprad Family Foundation for Entrepreneurship, Research and Charity (Grant No. 20170012). CW was additionally funded by the Swedish Research Council for Health and Welfare (Grant No. 2017-01451). The funders did not have any role in the design, collection, analysis, interpretation of data, writing the article, and decisions regarding it.

## Conflict of interest

The authors declare that the research was conducted in the absence of any commercial or financial relationships that could be construed as a potential conflict of interest.

## Publisher's note

All claims expressed in this article are solely those of the authors and do not necessarily represent those of their affiliated organizations, or those of the publisher, the editors and the reviewers. Any product that may be evaluated in this article, or claim that may be made by its manufacturer, is not guaranteed or endorsed by the publisher.
